# Empirical study of college students’ extracurricular reading preference by functional data analysis of the library book borrowing behavior

**DOI:** 10.1371/journal.pone.0297357

**Published:** 2024-01-26

**Authors:** Fan Zhang, Yuling Liu, Chao Song, Chun Yang, Shaoyong Hong

**Affiliations:** 1 The Library of Guangzhou Huashang College, Guangzhou, China; 2 School of Data Science, Guangzhou Huashang College, Guangzhou, China; 3 School of Accounting, Guangzhou Huashang College, Guangzhou, China; NIT Srinagar: National Institute of Technology Srinagar, INDIA

## Abstract

Library data contains many students’ reading records that reflect their general knowledge acquisition. The purpose of this study is to deeply mine the library book-borrowing data, with concerns on different book catalogues and properties to predict the students’ extracurricular interests. An intelligent computing framework is proposed by the fusion of a neural network architecture and a partial differential equations (PDE) function module. In model designs, the architecture is constructed as an adaptive learning backpropagation neural network (BPNN), with automatic tuning of its hyperparameters. The PDE module is embedded into the network structure to enhance the loss functions of each neural perceptron. For model evaluation, a novel comprehensive index is designed using the calculus of information entropy. Empirical experiments are conducted on a diverse and multimodal time-series dataset of library book borrowing records to demonstrate the effectiveness of the proposed methodology. Results validate that the proposed framework is capable of revealing the students’ extracurricular reading interests by processing related book borrowing records, and expected to be applied to “big data” analysis for a wide range of various libraries.

## 1. Introduction

Extracurricular learning is an important part of college students’ knowledge acquisition and largely affects students’ graduation, job search, and even future development [1]. Besides focusing on professional course learning, the extracurricular learning behavior of college students is gradually becoming a concern for college education management [2, 3]. Understanding the extracurricular learning preferences of college students can help predict the habits and status of college students’ reading habits at an early stage, which is engaged to prevent the bad consequences of students’ biased learning.

To date, there is no unified standard to define the connotation of extracurricular reading conceived to improve students’ personal comprehensive qualities. Different scholars hold different views. For instances, some researchers adopted the PISA 2009 definition of reading engagement to describe that the extracurricular reading is usually affected by the influences of reading time, reading quantity, and reading interest [4]. Extracurricular reading is validated to be in positive interaction with the students’ academic learning. However, excessive reading books that are not relevant to the main academics draw back the educational outcomes for college students, such as cumulated grades or at risk of failure in academic courses, based on students’ demographics, past test performance and course attending history [5]. Most of them show that past test performance is highly predictive of future success/failure and that a set of demographic characteristics achieves reasonably high predictive accuracy [6].

Accumulation of extracurricular knowledge will comprise advantages and some potential risk. Extracurricular books are very informative such as the developed/learned individual engagement taxonomy and completing some life-style informal courses [7, 8]. Students’ performance on specific problems are based on comprehensive knowledge and interaction logics from diverse curriculums. They response to the problems with concepts/skills that are not specified by teachers, but recorded in multi-field books [9]. The reading is much beneficial to broaden the range of students’ exposure to knowledges with daily increase of the reading time and reading amount, and the involvement of reading diversity [10, 11].

As a place for students learning, the campus library is a source site to study students’ extracurricular reading behavior. Objectively, every college library has a book access system is a one-way module that fully records the students’ personal information and their histories of borrowing books [12]. Then, the students’ learning and reading behaviors can be investigated by analyzing these recorded data. However, many students from different majors go to the library and check out a variety of different types of academical or extracurricular reading materials. The library has accumulated massive book borrowing and returning data, and also the staff regularly conducts descriptive statistical analysis of the data [13, 14]. Thus, the collation, selection and further analysis of the library data is a real big data problem. Aiming at big data and data mining problems in college library, college students’ book check-out data are deeply mined possibly by data fusion, behavior quantification, and performance prediction [15]. In recent years, quality education is on hot topic and is supported by the promotion of wide range extracurricular book readings is prevalent [16]. The development of big data and AI has facilitated the investigation of students’ learning and living habits and effectively improved the prediction accuracy and universality of data analytical results [17].

Based on students’ data on the digital campus, students’ extracurricular interests are learned in one way by data mining on the students’ book borrowing/check-out records. Data mining is the technique to establish a meaningful model for analyzing, studying and summarizing the latent rules that are originally hidden in the large amounts of complex data [18, 19]. By using the intelligent computing methods, the theory of statistics and the database technology, data mining requires to make classification tags in advance, which are a set of categories with different characteristics [20]. Most researchers have begun to use statistical methods and some intelligent principles to predict students’ behaviors in digital campus living, to make reasonable decisions to explore the internal connections of the data [21–23].

The recorded data in the library is now in a heterogeneous state of dynamic change as the university campus is in a progressively accelerating fast-paced lifestyle. These changes have significant implications for the performance of the book borrowing and returning data [24]. Simple linear statistical methods are adopted in most studies. But the single-threaded static classification of book check-out behavior by the distinguishment of category tags is not sufficient to interpret the student’s extracurricular preference. Due to the weak connection and sparse information of the recorded digital data, the traditional statistic methods are relatively simple in behavioral data quantification. They are not sufficient to identify the latent data properties, especially in the behavioral order’s quantification [25].

As the scale of behavior variation continues to grow, the current solutions for data processing become little able to optimize the library operations in terms of performance, cost, energy consumption, privacy, and security. Functional data analysis is a good solution to explain the comprehensive influence from diverse multi-variate big data mining problems [26]. The functional equation model leads to the fully extract the digital resources. In recent years, PDE has been utilized as the derivation of computational methods for the support to enhance applied statistics, machine learning and computation sciences [27–29].

Many social and natural phenomena and engineering problems can be described by partial differential equations (PDE), such as image processing and population distribution [30, 31]. The PDE models are able to fully interpret the deducing effects of each concerning factors in a data analytical manner [32]. Coincidentally, the book borrowing behavior is a multi-factor time series issue that carries many hybrid uncertainties to the data analytical model [33]. Actually, there has been little researches reporting that functional data analysis methods, especially the PDE, to study the college library data, in the aim to investigate the college students’ extracurricular preference.

With the development of AI technologies, intelligent computing methodologies have demonstrated their strong capability to overcome the bottlenecks in qualitative or quantitative analysis [34, 35]. On these bases, we proposed to build up an intelligent computing framework, to establish the data mining models for the research of students’ extracurricular interests based on the book reading habits in campus libraries. The frame work is in fusion of a backpropagation neural network (BPNN) architecture, an improved PDE function module, a unit of information entropy for comprehensive enhancement. These methods/algorithms are targeted to solve some abovementioned urgent hot challenges for data analysis of the library book borrowing records (See Table 1). In details, we designed a scalable tuning strategy for the network structure, and modified the PDE functional module to suit our model optimization process. the proposed methodology is used to extract potential information from the diverse and multimodal data, with the main concerns on the properties of different book catalogues, varied reading interests for genders, fusion quantification of students from different majors. The framework is able to extract hidden features from the library book borrowing data with the aim to analyze the college students’ non-academical interest.

**Table 1:** The confronting challenges and our planned solutions.

Challenges	Planned solution
Students’ performances are not all learned from academic classes. [11]	Quantitative analysis based on library data of extracurricular reading
Multiple factors influencing extracurricular reading [4]	Diverse multivariate model
No previous data analysis to investigate students’ reading interest by quantification modeling	Study of machine learning methods
Massive book borrowing and returning records [26]	Network-based feature selection
Extracurricular reading is interactive with academic learning. [5]	Fusion with a PDE function module
Library records are complex time-series data [33]	To design comprehensive indicators in aid with information entropy

In the future, the proposed modeling methodology is prospectively expected to be embedded into an IoT-clouded framework, enabling it to effectively process the large amount of big data collected from various college libraries across different locations.

## 2. Experimental data

The libraries have a rich collection of books in number and types. Students who make full use of these resources usually have better performance on academy and extracurriculum. The library book-borrowing system records all the book check-out information. For data mining task of this study, we mainly extracted the two parallel related tables: the book catalogue as well as the book check-out history; the gender information and the subject specialty of the borrowers.

**Fig 1:**
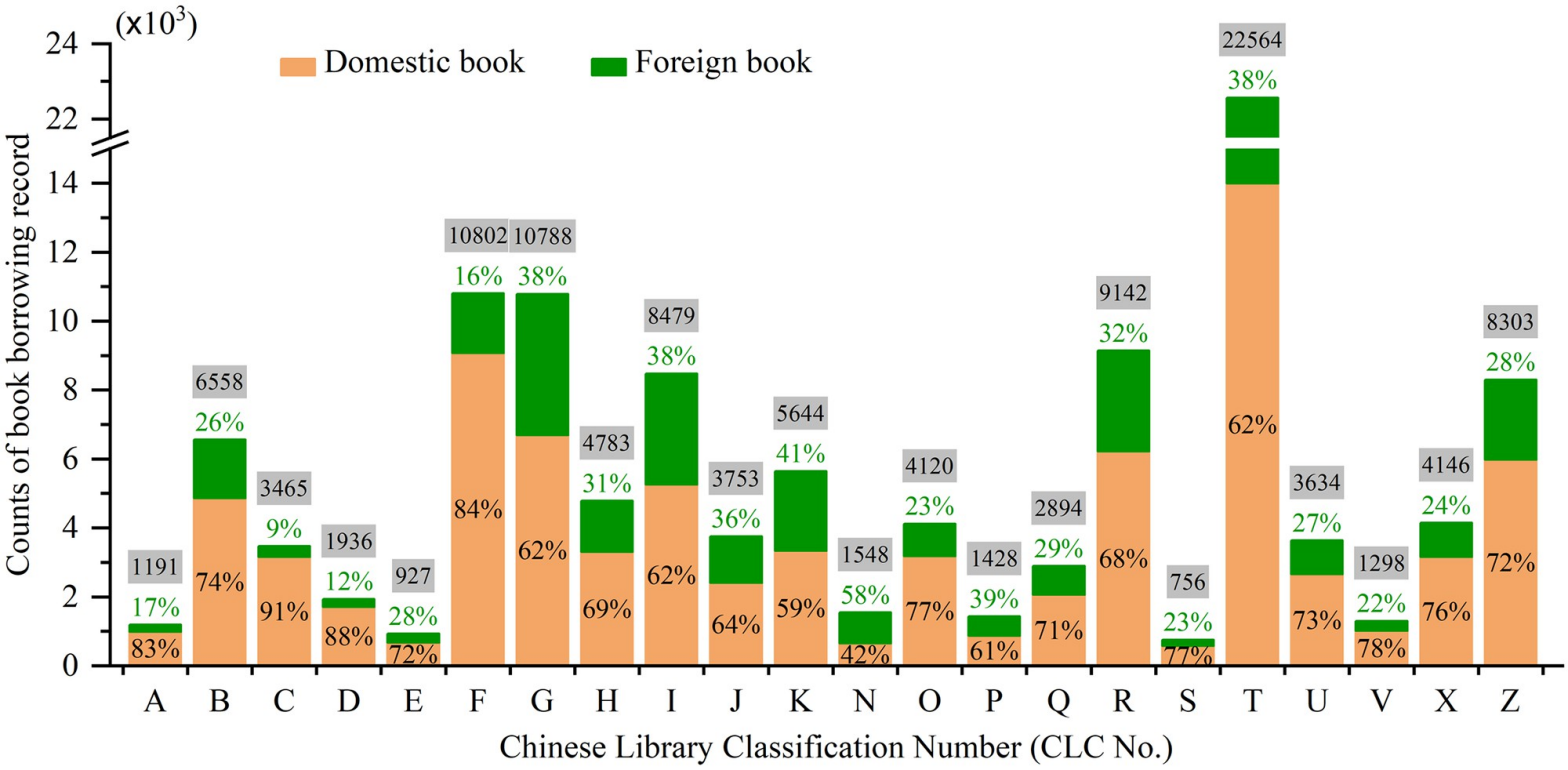
Counts of the domestic and foreign book borrowing records classified by CLC catalogue.

**Fig 2:**
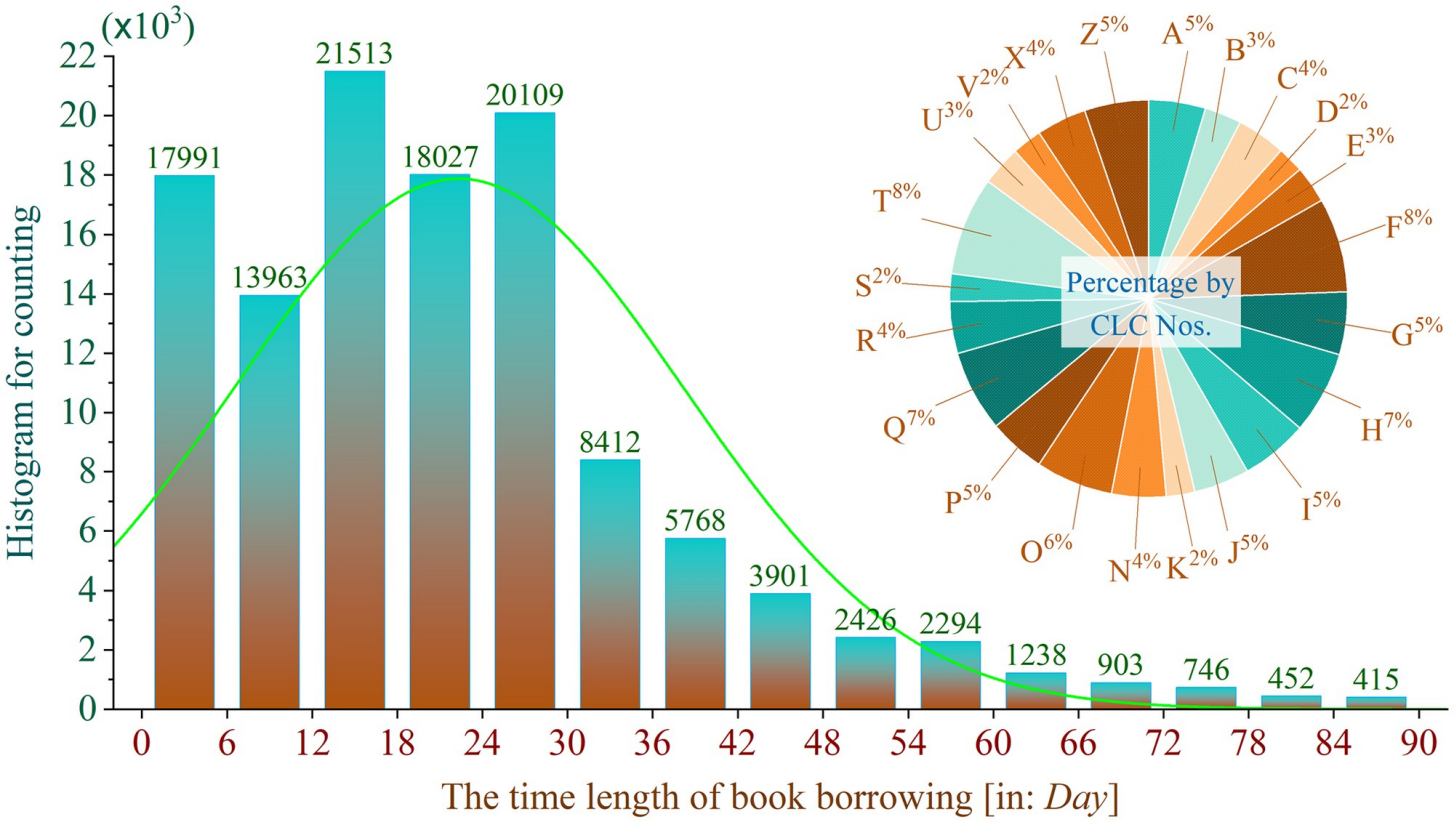
The histogram for the distribution of time length of book borrowing.

Concerning on the extracurricular learning, we sorted out the records of borrowing non-specialty-related books. There we have 118,159 book borrowing records. To get deep knowledge to the record data, basic statistical work is firstly done to the book check-out information, simply classified by the three indicators: the Chinese library classification number (CLC) catalogue, the distinguishment of domestic and foreign books (DDF) and the book borrowing time length (LEN). Resultantly, we have the amount counts of check-out records for each CLC number with DDF tags showed in Fig 1. Here we acknowledge that the students prefer to read domestic books than foreign book in a general ratio of ~7:3. The popular CLC numbers are T, F, G, R, I and Z (over 8,000 records for each), which represent the book classifications of *Engineering* (including *Mechanism*, *Electrics & Electronics*, *IT & Communication*, *Control & Automation*, etc.), *Economy*, *Cultural Education*, *Medicine & Health*, *Literature* and *The General*, respectively. Besides, we also have summarized the LEN data in groups of small separate ranges of every 6 days (See Fig 2). It is easily found that the LEN data shows a normal distribution with left skewness. The most frequent book check-out time length is within 30 days (i.e. almost in one month).

**Fig 3:**
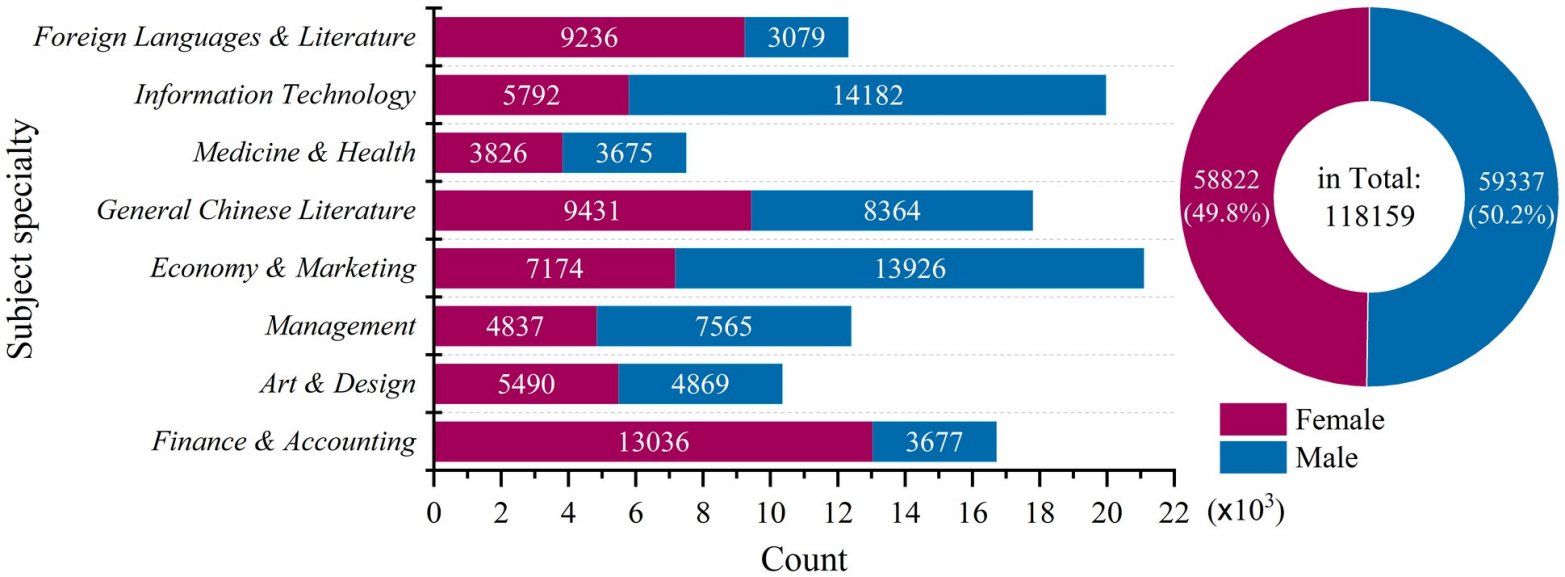
Counts of the book borrowing records based on the distribution of students’ information (in segments of subject specialties and by genders).

When focusing on the book borrowers’ personal information, we count the check-out records for different academic subject specialty (ASS) the students take and by the students’ gender distinction (GEN) (see Fig 3). We can see that the students who major in *Economy & Marketing*, *Information Technology*, *Finance & Accounting*, *General Chinese Literature* have the top book borrowing records. On the other hand, the book borrowing times by female and male students are almost the same.

Based on this statistical information, we establish the data mining models for the research of students’ extracurricular interests. An intelligent computing framework is built up by investigating the PDE improvement for functional data modeling.

## 3. Methodologies

### 3.1 Design of the full modeling framework

**Fig 4:**
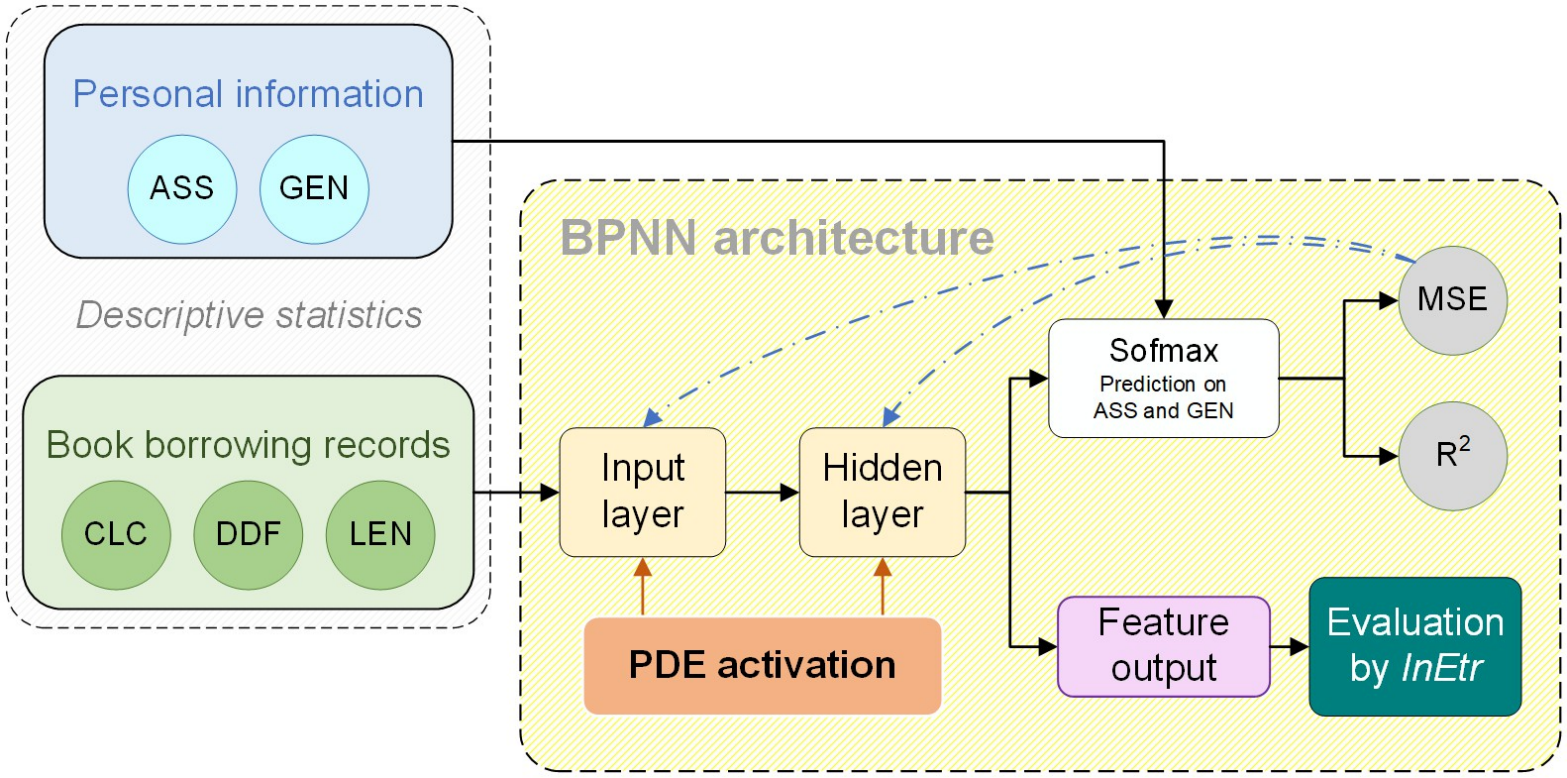
The full modeling framework by fusion of the BPNN, the improved PDE module and the *InEtr* enhancement.

Concerning on the extracurricular learning, the investigation of factors affecting students’ book borrowing is the premise and guarantee for the accurate attribution and quantification on non-academic interests. To this aim, we built up the intelligent computing framework. A general BPNN architecture is constructed. The PDE functional module is modified in algorithm, and applied in fusion for the activation of the network-extracted features. Because the book check-out data from the digital library system is a kind of multimodal data, the detail properties cannot solo clarify the behavior, but interact with each other. Thus, we employ the calculus of information entropy to generate a comprehensive indicator with targeted on the extracted features for model evaluation. The full architecture of the proposed model is built up for functional data analysis of the library book borrowing behavior (see Fig 4). Detail algorithmic designs of the BPNN architecture, the improved PDE function module and the calculus of information entropy are introduced in the following sections.

Quantification may be launched based on the framework. For evaluation, feature visualization and correlation analysis explore students’ book-borrowing models. The absolute value of Pearson’s correlation coefficient (*r*) exposes the correlation strength,

$$r=\frac{\sum \left(x_{i}-\bar{x}\right)\left(y_{i}-\bar{y}\right)}{\sqrt{\sum {\left(x_{i}-\bar{x}\right)}^{2}}\sqrt{\sum {\left(y_{i}-\bar{y}\right)}^{2}}},$$ (1)

where *x*_*i*_ and *y*_*i*_ represent two different features extracted by BPNN model.

Furthermore, accurate assessment of the model’s generalization ability requires indicators for the model’s performance measurement. The commonly used evaluation indicators are the mean square error (MSE) and the determination coefficient (R^2^), which are formulated as,

$$\text{MSE}=\frac{1}{n}\sum_{i=1}^{n}{\left(y_{i}^{\prime }-y_{i}\right)}^{2},$$ (2)

$${\text{R}}^{2}=1-\frac{\sum_{i=1}^{n}{\left(y_{i}^{\prime }-y_{i}\right)}^{2}}{\sum_{i=1}^{n}{\left(\bar{y}-y_{i}\right)}^{2}},$$ (3)

where $y_{i}^{\prime }$ and *y*_*i*_ are the model prediction value and the actual value of some quantified book check-out records; $\bar{y}$ is the mean value of {*y*_*i*_}. Generally speaking, R^2^ is a value ranged in [0, 1]; the larger R^2^ is, the better the model performs.

### 3.2 The BPNN architecture

A general BPNN structure is consist of one input layer, one hidden layer and one output layer. Each layer has a designated number of neural nodes, where the nodes take perceptron calculations on the basis of summation, activation under a threshold control [36]. The network mapping from the input layer straight through to the output layer will definitely obtain the model predictions on any preset target indicator [37]. The number of input nodes (*N*_in_) are set according to the sample size, the data properties are delivered to different nodes/perceptrons that are available to receive the data in multimodal types. Every perceptron has a set of hyperparameters including the batches of linking weights (denoted as *w*^(*k*)^ for the *k*-th perceptron), a summation operator (formulated as ∑∙) and an activation function (denoted as *f*(∙)). The detail implementation of BPNN is showed as the following procedures,

Step 1, Network initialization: The number of hidden nodes (*N*_hidden_) is set tunable as integers. The number of output nodes (*N*_out_) is also tunable for delivering the selected data features.
Step 2, The perceptron calculation: The following equation shows the calculation of each perceptron, no matter it is in the hidden layer or in the output layer.
$$h_{j}^{\left(k\right)}=f\left(\sum w_{ij}^{\left(k\right)}\cdot x_{i}+\theta_{j}^{\left(k\right)}\right)\;\text{for}\;\forall k\;\text{and}\;j=1,2\ldots$$ (4)
  where the subscripts *i* and *j* point to the *i*-th input node and the *j*-th output node of the perceptron; *θ* is the threshold defined in the summation calculation; $H^{\left(k\right)}=\left\{h_{j}^{\left(k\right)}\right\}$ represents output data feature of the *k*-th perceptron unit.
Step 3, The activation function *f*(∙) usually adopts the tanh or the ReLU function, namely,
$$f\left(x\right)=\left\{\begin{array}{l} \frac{e^{x}-e^{-x}}{e^{x}+e^{-x}}≜\text{tanh}\left(x\right),\\ \text{max}\left(0,x\right)≜\text{ReLU}\left(x\right). \end{array}\right.$$ (5)
Step 4, The transformed features observed from the output layer and used for the prediction of the network output value $\left\{y_{n}^{\prime }\right\}$.
Step 5, According to the predicted value and the known expected value {*y*_*n*_} of all samples, the error (*E* = {*e*_*n*_}) is calculated successively,
$$e_{n}=\left|y_{n}^{\prime }-y_{n}\right|,\;\text{for}\;n=1,2\ldots$$ (6)
Step 6, The error information is fed back to each perceptron for iterative network refinement, namely, *w*^(*k*)^ = {*w*_*ij*_}^(*k*)^ and *θ*^(*k*)^ = {*θ*_*j*_}^(*k*)^ are updated for the *k*-th perceptron as follows,
$$\left\{\begin{array}{l} w_{ij}=w_{ij}+\mu \cdot x_{ij}\cdot \left(1-h_{j}\right)\cdot ||E_{2}||,\\ \theta_{j}=\theta_{j}+\eta \cdot h_{j}\cdot ||E{\left|\right|}_{2}. \end{array}\right.$$ (7)
  where *μ* and *η* are the indices regarding the iterative learning rate.
Step 7, The neural network architecture is trained automatically by self-adaptively tuning the hyperparameters.

Technically, the error feedback iterative optimization developed by back propagation will not stop until the predictive error value reaches a specified limit or the iterations reaches the preset magnitude.

### 3.3 The improved PDE function module

PDE model is frequently applied in many industrial and engineering cases to describe the technical problems, such as image process, population distribution, traffic dispatching and some IoT sceneries [38–40]. The general form of PDE is defined as follows,

$$G\left(x,\nabla \varphi ,\nabla^{2}\varphi \right)=0$$ (8)

where *x* = (*x*_1_, *x*_2_… *x*_*n*_) is the independent variable and *x* ∈ *D*, *D* represents a pre-defined square domain of the real numbers, i.e. *D* ⊂ *R*^2^; *φ* is an undetermined function with solo dependence on *x*, namely, we have *φ* = *φ*(*x*); ∇ and ∇^2^ are the special operator to calculate the 1st-order and 2nd-order gradience, respectively.

In common sense, the differential problem defined as formula (8) can be solved only if it is defined with initial and boundary conditions. However, the phenomena of insufficient information and some practical instable factors probably lead to uncertain parameters during the modeling processes. Obviously, the conditions should come from the actual data records by technically extracting the features using the BPNN architectural framework. There we re-define the initial boundary PDE problem as follow,

$$\left\{\begin{array}{l} G\left(x,\nabla \phi ,\nabla^{2}\phi \right),\\ G{|}_{t=0},\;x\in D,\\ G{|}_{\Gamma },\;\;t>0, \end{array}\right.$$ (9)

where the function *ϕ* is a modified form of *φ*. Specifically, *ϕ* is of the novel definition as *ϕ* = *ϕ*(*x*, *p*), which is able to endorse batches of the BPNN linking weights as the hyperparameter *p* into the PDE module for fusion model tuning; *G*|_*t* = 0_ represents the initial case of *G* and *G*|_Γ_ shows the mappings limited on the boundary situation. Then, the optimization of the fusion model deems to alternatively find the minima of the sum of *G*(∙) for each input of the discrete data {*x*_*i*_|*i* = 1,2…*n*}.

We suppose the time series in the full interval of *t* = [0, *T*]. The full interval is divided into uniform grids with *t* equals to the gradually increasing integers. Base on the convolution quadrature generated by BDF3/BDF4 [41], the approximation of $G_{t}={G|}_{t=t_{n}}$ is described by

$$\frac{\text{d}G_{t}}{\text{d}t}=\frac{1}{t}\sum_{i=1}^{n}\left(u_{i}-iG_{t}\left(x_{i}\right)\right),$$ (10)

in which *u*_*i*_ is a dynamic weight variable as the time moves, which can be provided by data fitting at each time moment, namely to fit the derivation according to the following equation,

$$\frac{1}{t}\sum_{i=1}^{n}u_{i}x_{i}=\delta_{t}\left(\xi \right),$$ (11)

where $\delta_{t}\left(\xi \right)=\frac{1}{t}\sum_{\tau =1}^{t}\frac{1}{\tau }{\left(1-\xi \right)}^{\tau }$. Such that the discretization for the time-series updated data is accomplished. Therefore, the optimization of the BPNN in fusion with PDE refreshes its targeted loss function applied to the discretized data, which is formulated as

$$\begin{array}{c} {\text{min}}_{p}\;\sum_{i=1}^{n}G\left(x_{i},\nabla \phi \left(x,p\right),\nabla^{2}\phi \left(x,p\right)\right),\\ \text{s}.\text{t}.\;G{|}_{t=0}\;\text{and}\;G{|}_{\Gamma }. \end{array}$$ (12)

### 3.4 Fusion of BPNN and the improved PDE module

**Fig 5:**
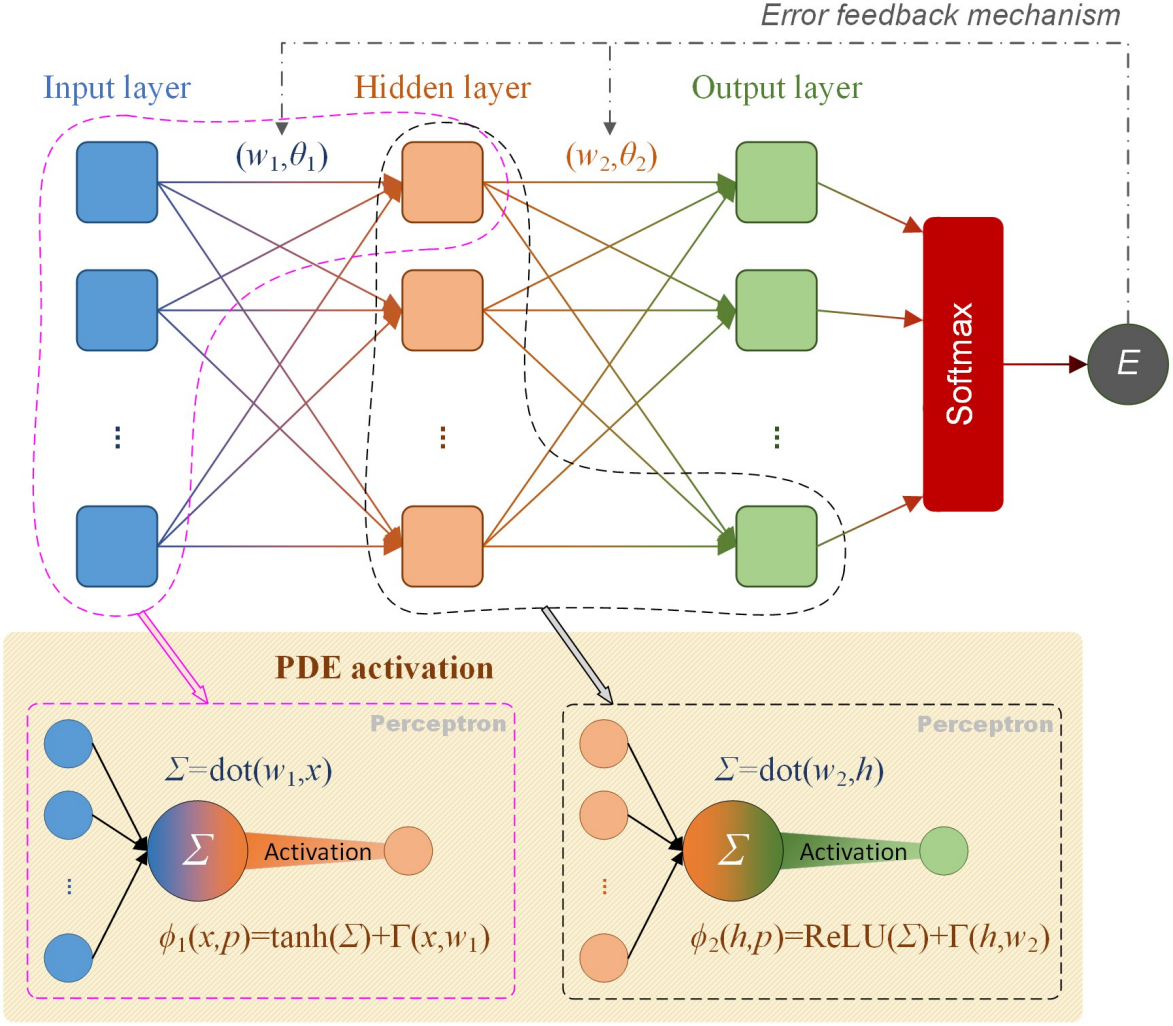
The designed BPNN architecture in fusion with the PDE module.

The fusion model of BPNN architecture with the improved PDE function is able to approximate any continuous nonlinear function with arbitrary precision. To specify the solution to formula (12), the model adopts the function *ϕ*(*x*, *p*) as the sum of the following two parts,

$$\phi \left(x,p\right)=f\left(net,x_{0}\right)+\Gamma \left(x,w\right),$$ (13)

where *f*(*net*, *x*_0_) is a fusional activation function endorsing the combination of the network transmission and PDE computation; *net* = *net*(*x*, *p*) is the output of BPNN with the tuning of its internal parameters; *x*_0_ = *x*|_*t* = 0_ represents the initial condition of the PDE option; and *Γ*(*x*, *w*) represents the boundary condition related to the input data as well as the network linking weithgts. In this way, the loss function in formula (9) can be solved accordingly. Consequently, we build up the fusion model of BPNN architecture with the improved PDE as the intelligent computing framework, see Fig 5, to extract hidden features from the college library recorded data.

### 3.5 The enhanced algorithm design by information entropy

As the network model is activated by the PDE functions with initial and boundary conditions, we take the library book borrowing records as time series for iterative optimization. Information entropy (*InEtr*) is able to quantify the behavior complexity with overcoming many drawbacks of the multimodal data [42].

The time series calculation of the information entropy is basically shown as the following,

$$InEtr=-\sum_{t=1}^{T}x\left(t\right)\cdot \text{log}\;x\left(t\right),\;t=1,2\ldots T,$$ (14)

where *x*(*t*) is the time series feature data at the *t*-th moment; *T* represents the total length of the time series. And *T* is divided into *m* segments; thus, the time segmental sequences is showed as

$$x\left(t\right)=\left\{t\left(i\right),t\left(i+1\right)\ldots t\left(T+i-m\right)\right\}.$$ (15)

In sequence, the segmental information entropy in time series is re-defied as

$$InEtr≝\text{log}\left(\sum_{i=1}^{T-m+1}cnt_{i}(r<r_{0})\right)-\text{log}\left(\sum_{j=i}^{T-m}cnt_{j}(r<r_{0})\right),$$ (16)

where *i* ∈ [1, *T* − *m*), and *cnt*_*i*_ (*r* < *r*_0_) is a counting function that actually counts in the *i*-th time series segment the number of the data features whose correlation coefficient is less than *r*_0_ (i.e. a preset Pearson correlation).

## 4. Model establishment and result discussions

### 4.1 Optimization of BPNN with tunable parameters

The 118,159 book borrowing records are input to the BPNN architecture for training to extract the features from the multimodal data. As the data refer to the two-line parallel multimodal properties of book catalogue and book check-out history (CLC, DDF, LEN) and borrowers’ personal information (ASS, GEN). For empirical studies, the data is input to the BPNN model with the aim to see the difference of book check-out behavior among the ASS and between the GEN. Thus, the CLC, DDF and LEN data are taken as the input to the network architecture, and ASS and GEN data as the comparative labels for the output. The BPNN model was trained with its weights automatically adjustive to the gradient optimizational ends. Each perceptron is designed to suit the discretized data, with the refreshed activation function applied with the PDE improvement.

**Fig 6:**
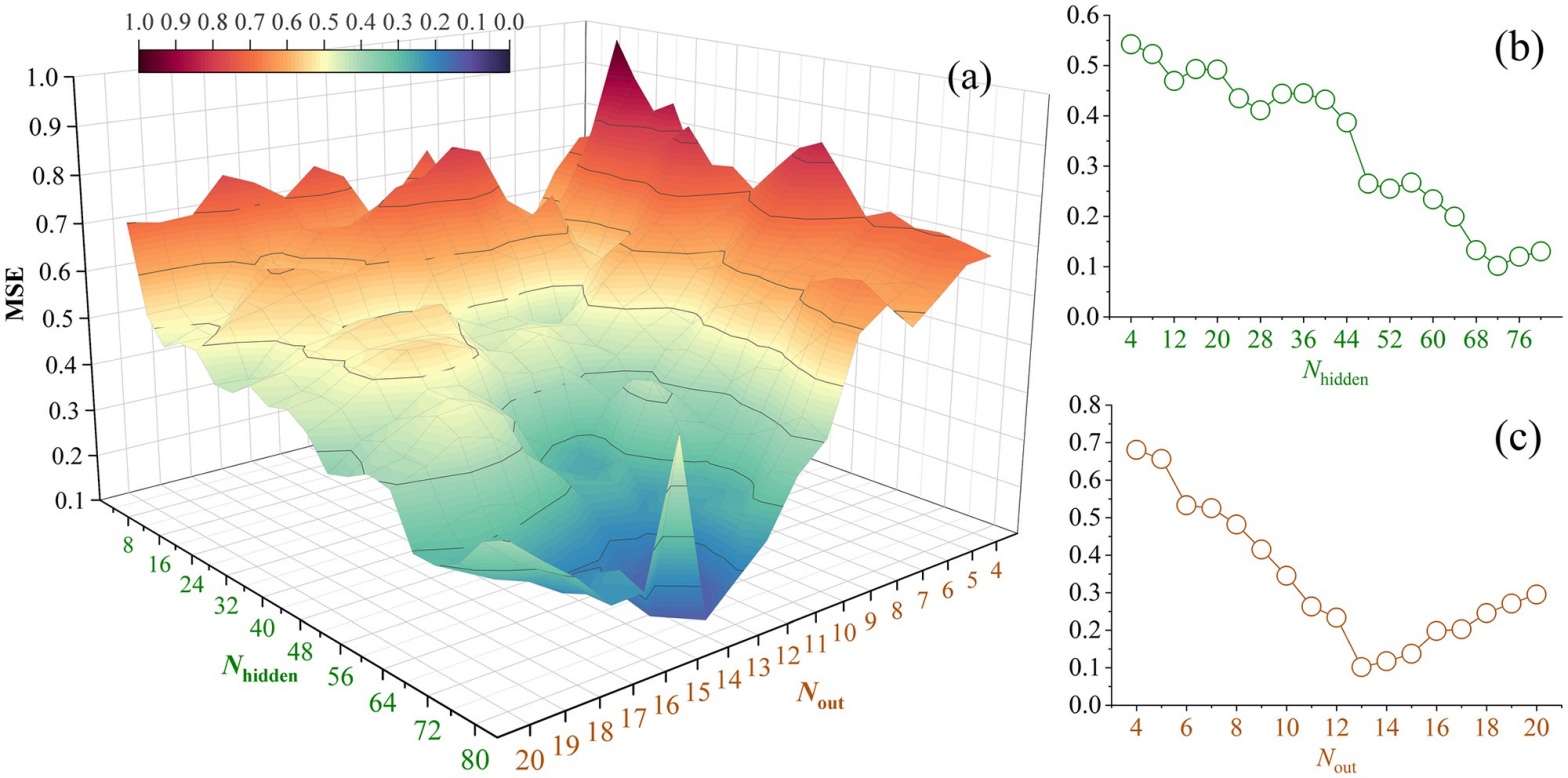
The prediction results of different structural BPNN models for quantitative analysis of the library book borrowing record data.

Different structural BPNN models are tested in the way of tuning *N*_hidden_ as from 4 to 80 in the step of 4 and tuning *N*_out_ as from 4 to 20. The capabilities of each model were evaluated according to the resultant MSE values. Fig 6 shows the training effects of different structural BPNN models under different values of *N*_hidden_ and *N*_out_, respectively. In sub-figure (a), the coloured surfaces represent the predictive results from each of the BPNN models corresponding to the designated values of *N*_hidden_ and *N*_out_. To find the optimal model, we produced the projection on both of the parametric axes under the rule of minimizing the MSE value. Thus, we have the sub-figure (b) showing the minimum MSE corresponding to each value of *N*_hidden_ for *N*_hidden_ = 4,8,12…80 (counting for 20 different numbers); and the sub-figure (c) depicting the minimum MSE corresponding to each value of *N*_out_ for *N*_out_ = 4,5,6…20 (counting for 17 numbers). It can be found from Fig 6 that the most optimal BPNN model is constructed with 72 hidden nodes and 13 output nodes. The corresponding minimum MSE is 0.102. Other than the best model, we can find some appreciable BPNN structures that are able to establish available models obtaining good quantification results close to the best model. They are identified built up by valuing *N*_hidden_ ∈ {68,72,76,80} in combination with *N*_out_ ∈ {13,14,15}.

### 4.2 Analysis of the information entropy for PDE module

**Fig 7:**
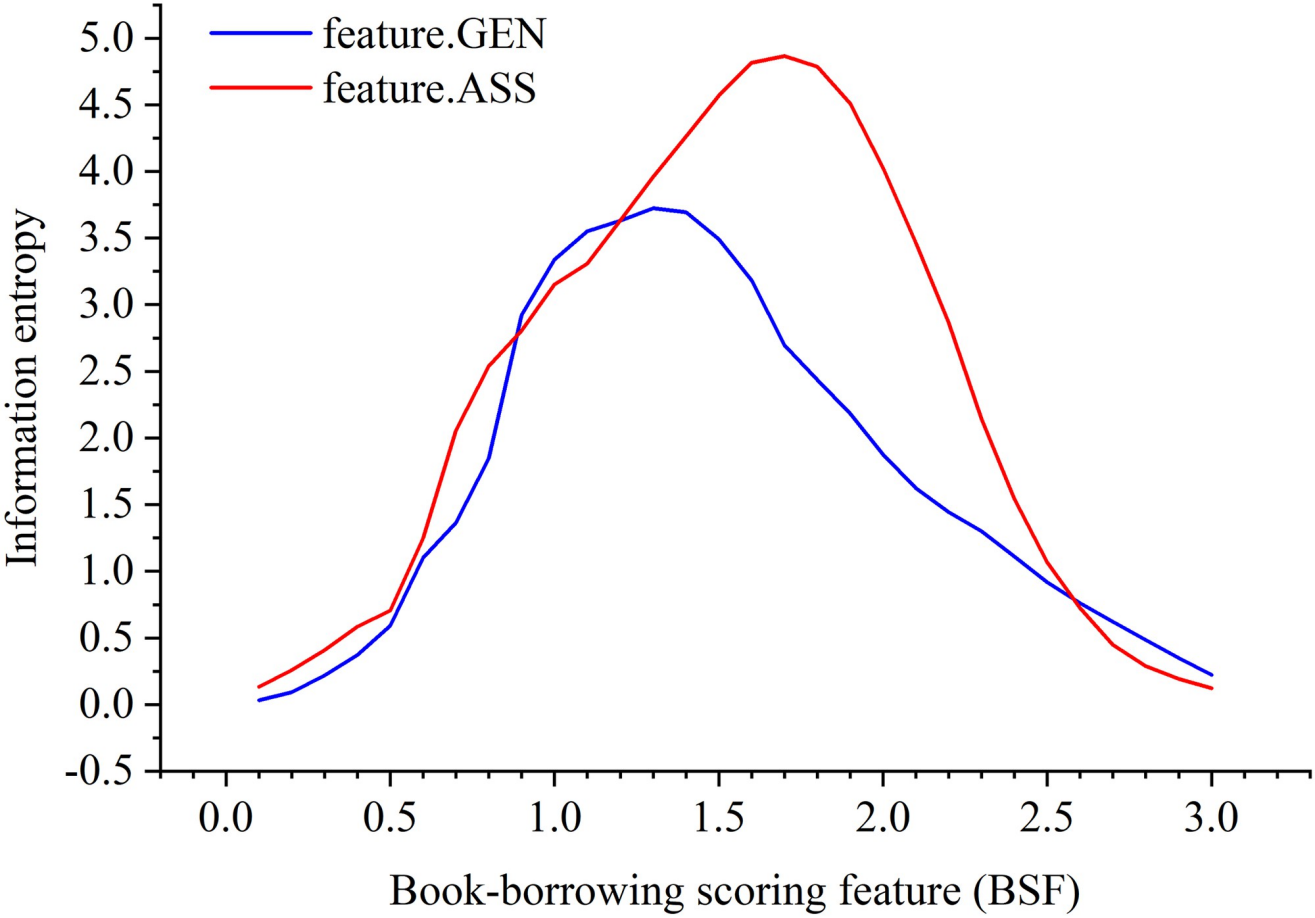
The BSF probability distribution on information entropy.

The three different indicators of CLC, DDF and LEN mainly reflect the book check-out information on students’ book reading behaviors. These indicators can be aggregated into a comprehensive index by weighting method to evaluate the indicator performance comprehensively. The comprehensive index is defined as the book-borrowing scoring feature (BSF). The calculation is as follows,

$${\text{BSF}}^{j}=\sum_{k=1}^{N_{hidden}}\alpha \cdot \phi \left({\text{CLC}}_{k}^{j},w\right)+\beta \cdot \phi \left({\text{DDF}}_{k}^{j},w\right)+\gamma \cdot \phi \left({\text{LEN}}_{k}^{j},w\right),$$ (17)

where *j* is the student’s serial number and *j* ∈ {1,2…118159}; *k* represents any BPNN extracted feature variable, *N*_*hidden*_ is the abovementioned number of hidden neurons. After the BSF calculation of behavioral complexity-related index, students’ approximate entropy in different score ranges is computed by formula (16). Fig 7 shows that the information entropy distribution of the BPNN extracted features of GEN and ASS are close to the normal distribution in BSF scores. This result indicates that the comprehensive indicator of BSF has significant differences to show the students’ book borrowing behaviors based on the features of the book check-out indicators of CLC, DDF and LEN.

### 4.3 Prediction results by the fusion model

The BPNN model in fusion with PDE improvement endorses the parallel indicators of the original recorded data (i.e. the CLC, DDF, LEN, ASS and GEN) as well as the comprehensive index for model optimization (i.e. the BSF). An ablation experiment is further launched to realize the combinations of the multimodal features be quantified to explore the modeling effects of the fusion model. Behavioral extracurricular-reading-related indicators are evaluated by the variation of student behavior’s information entropy, which is a behavioral complexity indicator.

**Fig 8:**
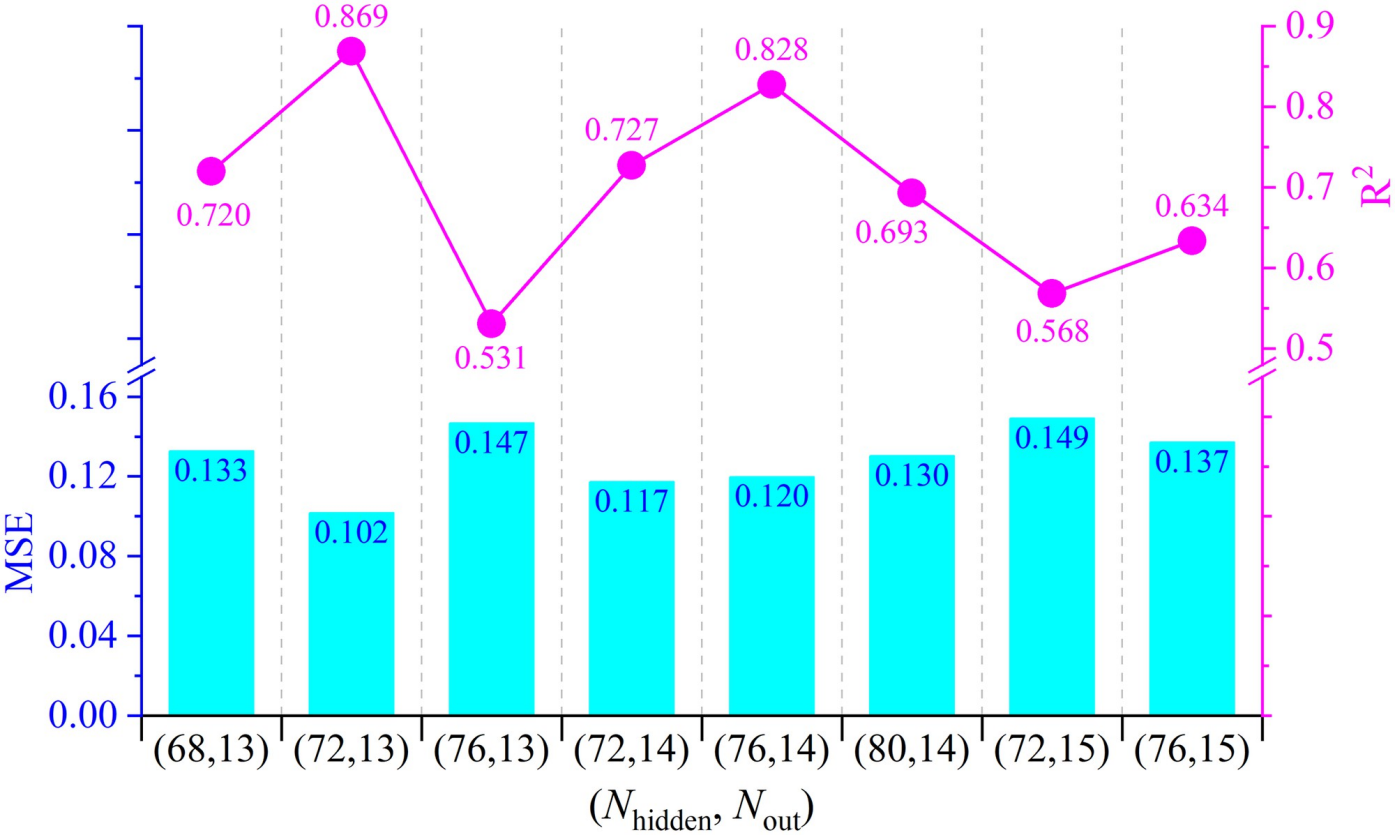
The predictive MSE and R^2^ for the available optimal 8 PDE-improved BPNN models.

With model optimization, the library book check-out records can recognize and predict the students’ non-academic learning behavior. A quantification model for the extracurricular learning customs is constructed for the PDE-improved BPNN model. The comprehensive index BSF is also added to the models, and the network parameters are re-trained. Then the models are evaluated by the quantitative MSE and the determination coefficient. Fig 8 shows the prediction results of the top 8 optimal models, in correspondence to the selected models in tuning of [*N*_*hidden*_, *N*_*out*_] as [68,13], [72,13], [76,13], [72,14], [76,14], [80,14], [72,15] and [76,15]. The best predicted R^2^ value reaches 0.869 at the maxima, and MSE goes to 0.102 at the minima. and the model predicted performance and actual performance showed a high correlation, the best prediction effect herein. Therefore, we conclude that the BPNN architecture is able to extract the data features that can well observe the students’ book borrowing behaviors, and the novel design of PDE improvement can better optimize the model.

Performances of the models have been improved with PDE gradient optimization, indicating that the proposed methodology has an essential contribution to accurate prediction models’ construction. The quantified indicators of students’ book borrowing in the library has significantly improved the prediction model’s performance. These results indicate that the extracurricular book check-out records play an essential role in reflecting the students’ non-academic reading behaviors.

## 5. Conclusions

The multimodal data in the college library book check-out system is investigated to observe the students’ extracurricular reading preference. The data is firstly analysis for descriptive statistics, focusing on the main indicators of CLC, DDF, LEN, ASS and GEN, of which the former three indicators refer to the book borrowing history, while the latter two represent the students’ properties of their own.

An intelligent computing framework is built up to extract hidden features from the college library recorded data, with the main concerns on fusion quantification, to analyze the college students’ non-academical interests. The framework is constructed based on the primarily BPNN architecture, with its self-adaptive hyperparameter tuning, in combination with the PDE functional model boosting for optimization. According to the experiment needs, the recorded data referring to the book check-out history is used to train the model to find the hidden features to explain the extracurricular learning behaviors, then to further predict the students’ properties by the automatic network training. The PDE function module is used to modify the network optimization loss function as a time series target, by using its initial and boundary conditions. Furthermore, the model indicator results in the experiment are compared to explore the quantified behavioral features’ necessity for prediction models’ construction. Experimental results show that the most optimized BPNN model predicts the students’ extracurricular learning customs with the lowest MSE of 0.102 accompanied by a high determination coefficient of 0.869. Simultaneously, we have shown out several appreciable models that are not the best one but have the close prediction to the best one.

We concluded that the BPNN architecture in fusion with the PDE functional module is feasible to establish a regular modeling and optimization system for processing the library data. The model prediction results reveal the methodology can extract features from the raw recorded book-borrowing data, so as to reveal the students’ extracurricular reading interests beyond their common academic learning behaviors. The proposed analytical methods and the modeling system are theoretically propriate for application in the “big data” scene. In future, we would try to promote the framework to deal with series of book check-out data from a wide range of different libraries.

## Supporting information

S1 File (ZIP)
